# High prevalence of sexual infection by human papillomavirus and *Chlamydia trachomatis* in sexually-active women from a large city in the Amazon region of Brazil

**DOI:** 10.1371/journal.pone.0270874

**Published:** 2022-07-18

**Authors:** Leonardo Miranda dos Santos, Josiellem Damasceno de Souza, Hilary Acha Mbakwa, Akim Felipe Santos Nobre, Rodrigo Covre Vieira, Stephen Francis Ferrari, Anderson Raiol Rodrigues, Edna Aoba Yassui Ishikawa, João Farias Guerreiro, Maísa Silva de Sousa

**Affiliations:** 1 Laboratory of Molecular and Cellular Biology, Tropical Medicine Center, Universidade Federal do Pará, Belém, Pará, Brazil; 2 Tropical Medicine Center, Universidade Federal do Pará, Belém, Pará, Brazil; 3 Institute of Biological Sciences, Universidade Federal do Pará, Belém, Pará, Brazil; 4 Graduation in Medicine, Universidade Federal do Pará, Belém, Pará, Brazil; 5 Department of Ecology, Universidade Federal de Sergipe, São Cristóvão, Brazil; Ruđer Bošković Institute, CROATIA

## Abstract

**Background:**

The Human Papillomavirus (HPV) and *Chlamydia trachomatis* are the most prevalent Sexually Transmitted Infections (STIs) worldwide, and are associated cervical cancer and pelvic inflammatory disease, respectively. However, 80% of women testing positive are asymptomatic. In the Amazon region, young women, in particular, are widely exposed to the infections and their consequences.

**Objectives:**

Determine the prevalence of sexual infection by HPV and *C*. *trachomatis* in young, sexually-active women treated at a university health program in a large city of the Brazilian Amazon region.

**Methods:**

We amplified the *L1 gene* of HPV. We amplified *ompA* gene of *C*. *trachomatis* by nested PCR, and the study participants filled in a questionnaire on their social, epidemiological, and reproductive health characteristics. The data were analyzed using the Odds Ratio, to evaluate the degree of association of these variables with the observed infections.

**Results:**

The prevalence of infection by HPV was 15.5% (47/303). This infection was recorded in 32.2% of the women of less than 25 years of age (OR:3.02 [CI95%] = 1.32–6.92; *p* = 0.014), 17.9% of the single women (OR: 2.41 [CI95%] = 1.22–4.75; *p* = 0.014), 23.8% of the women that reported having first sexual intercourse at less than 15 years of age (OR: 2.22 [CI95%] = 1.16–4.23; *p* = 0.021), 20% of those that reported having had more than one sexual partner during their lifetime (OR: 3.83 [CI95%] = 1.56–9.37; *p* = 0.003), and in 28.3% that use oral contraceptives (CI95% = 1.33–5.43; *p* = 0.008). The prevalence of sexual infection by *C*. *trachomatis* was 4.6% (14/303), and this bacterium was present in 16.1% of the young women of less than 25 years of age (OR: 2.86 [CI95%] = 1.33–5.43; *p* = 0.008).

**Conclusions:**

We found a high prevalence of HPV in young, unmarried women who started their sex lives early, who had several sexual partners in their lives and who used oral contraceptives. The prevalence of *C*. *trachomatis* was high only in young women. Our data are in accordance with other studies in Brazil and in the world and may serve to base the formulation of diagnostic and screening measures for these infections in women in the Amazon.

## Introduction

Sexually Transmitted Infections (STI) caused by the Human Papillomavirus (HPV) and *Chlamydia trachomatis* are the most prevalent infections of this type, worldwide, although approximately 80% of the cases may not develop signs or symptoms of disease. These infections nevertheless have a major impact on women’s reproductive health, given that cervical infection by HPV may be related to the appearance of low (LSIL) or high grade (HSIL) intraepithelial squamous lesions, and cervical cancer [1, 2]. Infections of the genital tract by *C*. *trachomatis* are also associated with Pelvic Inflammatory Disease (PID), miscarriage, and avoidable infertility [3, 4]. The Human Papillomavirus is a virus with enveloped DNA that presents tropism to epithelial cells, and infects the skin and mucous membrane of the oral and anogenital regions. Based on a similarity of up to 89% of the *L1* gene, the HPV can be divided into types classified as either "high risk" (HPVs 16, 18, 31, 33, 35, 39, 45, 51, 52, 56, 58, and 59) and “probably” or “possibly” oncogenic, that is, HPVs 26, 53, 66, 67, 68, 70, 73, and 82 [5] Infection by high risk HPV is associated with 100% of malignant cervical tumors and 83.3% of malignant anal tumors, however, only the persistence of infection by high-risk HPV types is associated with tumor development [6].

*Chlamydia trachomatis* is a bacterium with obligatory intracellular tropism, due to its lack of enzymes for the metabolization of Adenosine Triphosphate, or ATP [7]. The analysis of the genetic profile of this micro-organism, based on four variable domains of the *ompA* gene, which codifies the principal protein of the Mitochondrial Outer Membrane Permeabilization (MOMP) of *C*. *trachomatis*, permits the classification of 19 genotypes that are related to three different clinical conditions. Genotypes A, B, Ba, and C are related to trachoma [8], while genotypes L1, L2, L2a, and L3 are related to invasive and systemic infections known as venereal lymphogranuloma [9]. Genotypes D, Da, E, F, G, Ga, H, Ia, J, and K are associated with asymptomatic and non-invasive STIs [10]. The World Health Organization (WHO) estimates that approximately 311,000 deaths are caused annually by cervical cancer associated with infections by HPV and *C*. *trachomatis*, which represent a drain of 517 million dollars on the resources of public health systems [11]. A number of developed countries have implemented public programs for the monitoring and immunization of HPV [12, 13] and the monitoring of sexual infections by *C*. *trachomatis* [14, 15]. In Brazil, the National HPV Vaccination Program for children of under 11 years of age [16] and Papanicolau smear test are standard strategies for the monitoring of cervical cancer. However, Brazil lacks an official molecular monitoring system of HPV infection, and sexual infection by *C*. *trachomatis* is not included in the National DST/AIDS Program [17]. This scenario contributes to the lack of reliable data on the real situation of the epidemiologies in the population of young women. The meta-analysis of Colpani et al (2020) [18] nevertheless showed that the prevalence of sexual infection by HPV in young women is 25.41%, while rates of sexual infection by *C*. *trachomatis* vary from 1.8% to 20.5% [19–28].

The urban centers of the Brazilian Amazon region have precarious social, economic, and epidemiological conditions, which contribute to the exposure of the resident female populations to STI, which is reinforced by the generally inadequate public health services, which contribute to the spread of infections by HPV and *C*. *trachomatis* [29–31]. In the present stud, we verified the prevalence of sexual infection by HPV and *C*. *trachomatis*, sexually active adult females treated at a university health program in a large city of the Brazilian Amazon region.

## Methods

### Study population and data collection

This cross-sectional study was conducted between January 2017 and December 2017. The target population was composed of women (n = 303) from the city of Belém, in Pará State, in the Amazon region of Brazil, who received gynecological care through an extension program at the public Federal University of Pará. These individuals were invited spontaneously to participate in the present study after providing informed consent.

We investigated a non-probabilistic, intentional, and conventional sample, composed of women of between 18 and 83 years of age, who had already started their sexual life, who had either never had a Pap smear or who had last had one more than one year prior to the study. Exclusion criteria were pregnancy, menstruation, and not wishing to participate in the study or not signing the informed consent form. The participants were asked to provide answers to a questionnaire and were made aware of the importance of providing reliable answers, in order to minimize possible bias in the study. The following set of variables was investigated: age, conjugal status, education, age at fist sexual intercourse, lifetime number of sexual partners, use of condoms, use of oral contraceptives, and cytological parameters.

Cervical secretions were collected during routine pelvic examinations using an cervical brush, and the samples were stored in cryogenic tubes containing 1 ml Tris-EDTA buffer (TE) [10 mM Tris-HCl pH 8.5; 1 mM EDTA] at a temperature of -20°C prior to testing.

### Ethics statement

This study was approved by the ethics committee of the Center of Tropical Medicine (NMT) at the Federal University of Pará (UFPA) under protocol number 1.218.417. In this study we did not include participants less than 18 years of age. Free and informed consent for participation in the study was obtained in writing prior to the collection of the samples and the epidemiological data. All the data were analyzed with complete anonymity.

### Extraction of the DNA

The DNA was extracted using a pureLink Genomic DNA Purification kit (Invitrogen, Carlsbad, CA, USA) according to the manufacturer’s instructions and stored at -20°C until analysis. A Polymerase Chain Reaction (PCR) of the PCR of the *B-globin* gene was performed before the detection of both the HPV and the *C*. *trachomatis*.

### Detection of the *L1* gene of HPV

The detection of sexual infection by HPV occurred through the amplification of the 449pb-458pb region of the HPV L1 gene. This gene belongs to a degenerated region that allows the identification of all types of mucosal HPV. Was amplified using the degenerate primers MY09 5’-GTCCMAARGGAWACTGATC-3’ and MY11 5’-GCMCAGGGWCATAAYAATGG-3’, with the following correspondents: M = A + C, R = A + G, W = A + T, Y = C + T [31]. This amplification generates a fragment of 450 base pairs (bps), and is based on 10 pmol of each primer, 1.0μL of sterile water, 5.0 μL of Go Taq Green Master mix, and 2 μL of genomic DNA, with a final volume of 10 μL. The positive control was a positive sample and amplified earlier. Was added to each reaction to evaluate the suitability of the reactions, with sterile water (UltraPure DNase / RNase-Free Distilled Water, Invitrogen, USA) being used as a negative control, to verify the possible contamination of the reagents. Subsequently, the 200.0 μL microtubes containing the reagents were transferred to an MJ96G thermocycler (Biocycler), where the samples were amplified after initial denaturation at 95°C for 4 minutes, followed by 35 cycles of denaturation at 95°C for 45s, hybridization of the primers at 55°C for 45s, and extension at 72°C for 45s, with a final extension at 72°C for 8 minutes. The amplified products were visualized by electrophoresis in 1% agarose gel with 0.5 mg/mL of ethidium bromide.

### Cytological analysis

The collection of the endocervical secretion sample was performed employing the Papanicolaou test, in which we used an endocervical brush, Ayres spatula, and a flat edge blade. We scraped the ectocervix and endocervix. The material was stretched on a glass slide with a frosted edge duly identified with the standard registration and fixed with 96% alcohol. The staining was performed according to the usual Papanicolaou stain, which consists of the use of Harris hematoxylin, Orange G and EA-36. After staining, the slide was analyzed by light microscopy. Smears without atypia were classified as normal, nonspecific inflammatory smears and caused by a microorganism. Atypical cells of indeterminate significance were divided according to their place of origin: glandular or squamous, AGG or ASC, respectively. Cells of squamous origin were also subdivided into lesions into Low-Grade intraepithelial lesion (LSIL, comprising cytopathic effect by HPV and CIN I), High-grade intraepithelial lesion (HSIL, comprising CIN II and CIN III) and Atypical Squamous Cells” (ASCs). High-grade lesion where micro invasion and carcinoma cannot be excluded invasive epidermoid. Glandular lesions were divided into Adenocarcinoma in Situ (AIS) and invasive adenocarcinoma. The results of the conventional cytology were classified using the Bethesda terminology [32].

### Detection of the *ompA* gene of *C*. *trachomatis*

*Chlamydia trachomatis* was amplified using a nested PCR protocol modified by Jalal et al. (2007) [33], which produced a sequence of 394 bps of the *ompA* gene of *C*. *trachomatis*. The first reaction used 6.0 μL of GoTaq Green Master mix (Promega, Madison, WI, USA), 0.5 μL (containing 20 pmol/μL of each primer) of the primers P1 (A) (5’GACTTTGTTTTCGACCGTGTT-3’) and P2 (5’AGCRTATTGGAAAGAAGCBCCTAA-3’), 2 μL of the genomic DNA, and 3 μL of sterile water for a final volume of 12 μL. The second reaction used 0.5 μL of the solution of the first reaction, 6.0 μL of *Go* Taq Green Master mix (Promega, Madison, WI, USA), 4.5 μL of sterile water, and 0.5 μL (20 pmol/μL) of the primers P3 (5’-AAACWGATGTGAATAAAGARTT-3̕) and P4 (5’-TCCCASARAGCTGCDCGAGC-3̕). In both steps of the nested PCR, negative and positive controls were used to optimize the results. Initial activation was conducted at 95°C in both stages of the PCR, but whereas this temperature was maintained for 5 minutes in the first stage, it was maintained for only 1 minute in the second stage. This activation was followed by 35 cycles of denaturation at 94°C for 40 s, annealing at 54°C for 30 s, and extension at 72°C for 90 s, with a final extension step at 72°C for 7 min. The amplified products were visualized by electrophoresis in 1% agarose gel with 0.5 mg/mL of ethidium bromide.

### Statistical analysis

The data were analyzed using the Statistical Package for Social Sciences (SPSS) version 21.0 (SPSS, Chicago, Illinois, EUA). The Odds Ratio test was used to examine the degree of association between the prevalence of infections by HPV and *C*. *trachomatis* and the different variables. The 95% confidence interval (CI) was calculated for all comparisons, and a *p =* 0.05 significance level was considered for all the analyses.

## Results

The ages of participants of the present study ranged from 18 to 82 years. The median (Interquartile Range) age was 44.0 (34.0–54.0) years. A large proportion (89.8%) of the participants were more than 25 years old. Overall, 62.4% of the participants were single, 60.0% had more than 8 years of education, and 72.3% reported having initiated sexual activity at less than 15 years. In addition, 67.7% of the participants had had more than one sexual partner during their lifetime, 64.7% reported not using condoms regularly during sexual intercourse, 82.5% did not use oral contraceptives, and 91.7% had a normal cytological examination.

The total prevalence of HPV infection was 15.5% (47/303), although the prevalence increased significantly to 32.2% of the women aged less than or equal to 25 years of age (OR:3.02 [CI95%] = 1.32–6.92; *p* = 0.014), 17.9% of the single women (OR: 2.41 [CI95%] = 1.22–4.75; *p* = 0.014), 23.8% of the women that reported having first sexual intercourse at less than 15 years of age (OR: 2.22 [CI95%] = 1.16–4.23; *p* = 0.021), 20.0% of those that reported having more than one sexual partner over their lifetime (OR: 3.83 [CI95%] = 1.56–9.37; *p* = 0.003), and 28.0% (OR: 2.86 [CI95%] = 1.33–5.43; *p* = 0.008) of the women that use use contraceptives (Table 1).

**Table 1:** Social, epidemiological, and reproductive health variables characteristics of infection by *Human papillomavirus* and *C*. *trachomatis* in sexually active women from Belém, Amazon region of Brazil.

Social Variables			Total (n = 303)		HPV positive 15.5% (47/303)		OR	CI 95%	*p-value*	*C*. *trachomatis* positive 4.6% (14/303)		OR	CI 95%	*p-value*
			N	%	N	%				N	%			
	Age (years)^a^	≤25	31	10.2	10	32.2	3.02	1.32–6.92	**0.014** *	5	16.1	5.61	1.75–18.01	0.005*
		>25	272	89,8	37	13.6				9	3.3			
	Conjugal status^a^	Single	189	62.4	34	17.9	2.41	1.22–4.75	**0.014** *	10	5.2	1.53	0.47–5.01	0.664
		Married	114	37.6	13	11.4				4	3.5			
	Education (years)^a^	>8	182	60	33	18.1	1.69	0.86–3.31	0.166	7	3.8	1.55	0.53–4.55	0.592
		≤8	121	40	14	11.5				7	5.7			
Epidemiological variables	Age at fist sexual intercourse (years)^b^	≤15	84	27.7	20	23.8	2.22	1.16–4.23	**0.021** *	2	2.3	0.42	0.09–1.92	0.389
		>15	219	72.3	27	12.3				12	5.4			
	Sexual partners in the life^b^	1	98	32.3	6	6.1	3.83	1.56–9.37	**0.003** *	4	4	0.82	0.26–2.71	0.986
		>1	205	67.7	41	20				10	4.8			
	Condom use^a^ ^b^^-^	Yes	107	35.3	17	15.8	1.04	0.54–1.99	0.974	5	4.6	1.01	0.33–3.12	0.799
		No	196	64.7	30	15.3				9	4.5			
Reproductive health variables	Oral contraceptive^a^	Yes	53	17.5	15	28.3	2.68	1.33–5.43	**0.008** *	1	1.8	0.35	0.04–2.73	0.491
		No	250	82.5	32	12.8				13	5.2			
	Cytological alterations^c^	Yes	25	8.3	7	28	2.31	0.90–5.89	0.130	3	12	3.30	0.85–12.75	0.181
		No	278	91.7	40	14.4				11	4			

OR: *Odds Ratio*: variables adjusted to each other in each group—bivariate analysis. 95% CI: 95% Confidence Interval.

*: Statistically significant *p-value*.

^a^:Current variables.

^b^:Anamnesis variables.

^c^: Cytological analysis

The overall prevalence of sexual infection by *C*. *trachomatis* was 4.6% (14/303), increasing significantly (OR: 5.16 [CI95%] = 1.75–18.01; *p* = 0.005) to 16.1% in the young women aged less than or equal to 25 years of age. Co-infection between HPV and *C*. *trachomatis* was recorded in only four cases.

Prevalences of HPV and *C*. *trachomatis* infection were 28% and 12%, respectively, in women who presented cytological alterations. No significant prevalence of these infections was identified in women who presented cytological alterations.

## Discussion

The total prevalence of HPV infection was 15.5% (47/303), although the prevalence increased significantly to 32.2% in women aged 25 or less, single women, those who started sexual activity at a young age and who reported using oral contraceptives, as indicated in Table 1.

Sexual infection by HPV is the world’s most common STI. When undetected and not treated early, this infection can lead to lesions of the uterus and cancer, however, only the persistence of high-risk HPV is associated with carcinogenesis and that the majority of virus infections are self-resolving [1, 2, 6]. Infections by multiple HPV genotypes are common in northern Brazil [34–37].

The present study identified an increase of more than twice the chances of infection in individuals who started sexual activity at the age of 15, an increase of more than twice in single women and more than three times greater in women who had more than a sexual partner instead of their lifetime. These findings are consistent with the results of previous studies in different regions of the world, which have shown an association between HPV infection and higher risk sexual behaviors, such as precocious sexual activity and multiple sexual partners [18, 38, 39].

One other important finding in the present study was the increase in infection in women that use oral contraceptives, a group that presented a 2.7 times greater risk of infection in comparison with the group that does not use this type of contraceptive. The use of oral contraceptives is known to be related to an increased risk of HPV infection, as well as the development of cervical adenocarcinoma [40].

Sophisticated techniques of nucleotide amplification and genotyping have been adopted in official HPV screening programs and the prevention of cervical cancer in primary and secondary healthcare in the United States [12] and Europe [13]. In Brazil, the prevention of cervical cancer is based on programs of vaccination for the tetravalent immunization of children of less than 11 years of age [16] and cytological screening using the Pap smear test [17]. Although the Pap smear is a low-cost and high-sensitivity procedure a recent proposal for DNA-HPV screening in a small town in the Brazilian state of São Paulo obtained promising results [41]. Our population is adult and none of the participants in this study reported having been vaccinated against HPV, as the vaccination campaign only started in 2014 and was targeted at pre-adolescent people.

The overall prevalence of sexual infection by *C*. *trachomatis* was 4.6% (14/303), increasing significantly (OR: 5.16 [CI95%] = 1.75–18.01; p = 0.005) to 16.1% in women aged 25 or less, as indicated in Table 1. Other studies in the Amazon report that young age increases the chances of a sexual infection by *C*. *trachomatis* in women, and that this situation may be a consequence of other social conditioning factors that were not investigated in this study, such as poverty, sexual exploitation of children and adolescents and teenage pregnancy [42, 43].

While relatively few studies have focused specifically on sexual infection by *C*. *trachomatis* in the Amazon region, the results of the present study are consistent with most of the previous findings. High rates of infection have been recorded in young Amazonian women, including a rate of 11.9% in university students [22], 18% in parturients [21], 14.3% in young riverside dwellers on Marajó Island [23], 20.5% in women from the Tapajós region in the state of Pará [24], and 18% in pregnant women in the state of Amazonas [24].

The present study adopted a gold standard, which facilitated the early diagnosis of sexual infection by *C*. *trachomatis* and was clearly a valuable tool for the screening, as observed in the screening programs adopted in the United States [14], and Australia [15]. It is important to note, however, that many Amazonian populations are distributed in remote communities that are poorly or irregularly served by public health programs [23, 24, 26, 30].

Previous studies in Brazil have obtained similar results, in particular that young women are disproportionately vulnerable to sexual infection by *C*. *trachomatis*, with a prevalence of 11.5% in the Brazilian Midwest [27], 6.8% in the southern region [28], 12.3% in the Northeast [44], and 1.8% in the Southeast [20]. The findings of the present study are also consistent with those of other regions of the world, where high rates of infection have been recorded for both HPV [45] and *C*. *trachomatis* [11, 46] in the population of young adult women, of less than 25 years of age.

The relatively small sample size is the principal limitation of the present study, especially considering the ample spectrum of the populations found in Amazonian cities, together with possible social biases in the responses of the women to the questionnaires. The present study nevertheless represents a preliminary stage in a wider project focusing on different populations in the Brazilian Amazon region, which intends to identify potential indicators of these infections. The results of the present study nevertheless provide important preliminary insights into the epidemiological patterns of sexual infections byHPV and *C*. *trachomatis* in women from the Amazon region. More studies are needed to verify possible risk factors and epidemiological indicators of these STIs in Amazonian populations.

## Conclusions

We found a high prevalence of HPV in young, unmarried women who started their sex lives early, who had several sexual partners in their lives and who used oral contraceptives. The prevalence of *C*. *trachomatis* was high only in young women. Our data are in accordance with other studies in Brazil and in the world and may serve to base the formulation of diagnostic and screening measures for these infections in women in the Amazon.

## Supporting information

S1 File (DOCX)

S2 File (DOCX)

S3 File (DOCX)

S1 Dataset (XLSX)
