# Supplementary material for: High prevalence of sexual infection by human papillomavirus and Chlamydia trachomatis in sexually-active women from a large city in the Amazon region of Brazil
Source: PLoS One. 2022 Jul 18;17(7):e0270874. doi: 10.1371/journal.pone.0270874 (PMC9292084; doi:10.1371/journal.pone.0270874)
Supplement: S1 File — (DOCX) [file pone.0270874.s001.docx]

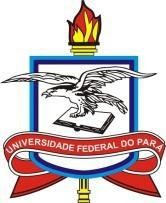


# SERVIÇO PÚBLICO FEDERAL UNIVERSIDADE FEDERAL DO PARÁ NÚCLEO DE MEDICINA TROPICAL

PROGRAMA DE PÓS-GRADUAÇÃO EM DOENÇAS TROPICAIS

LABORATÓRIO DE CITOPATOLOGIA

PROJETO DE EXTENSÃO: PREVENÇÃO DO CÂNCER DE COLO DO ÚTERO

LÂMINA:

COLETADORA:

DATA DA COLETA: / / DATA DA ENTREGA: / /

NOME: TELEFONE-

ENDEREÇO: EMAIL

ESTADO CIVIL ( ) Solteira ( ) Casada IDADE: ANOS PROFISSÃO/OCUPAÇÃO:

**Morou em outro município a menos de 3 anos?** ( ) Não ( ) Sim Qual?

**Renda familiar (salários mínimos):** ( ) menos de 1 ( )1 a 3 ( ) 4 a 6 ( ) 7 a 10 ( ) Mais de dez

**NIVEL DE INSTRUÇÃO**: ( ) NÃO FREQUENTOU ESCOLA ( ) < 8 ANOS ( ) > 8 ANOS ( ) SUPERIOR COMPLETO ( ) SUPERIOR INCOMPLETO **CURSO: PERÍODO: EDUCAÇÃO BÁSICA:** ( ) ESCOLA PÚBLICA ( ) ESCOLA PARTICULAR

**RENDA FAMILIAR**: **Consome bebida alcoólica?** ( ) Sempre ( ) Ocasionalmente ( ) Nunca

**Fumante**: ( ) Não ( ) Sim, quantos? Dia / Semana

Data da primeira menstruação: / / Data da última menstruação: / /

**Idade da primeira menstruação: anos Menstruação é regular**? ( ) Não ( ) Sim

Idade da primeira relação sexual:

Nº de parceiros DESDE o início da vida sexual:

anos

**e no ULTIMO ANO**: Data da última relação

sexual: / / usou preservativo nesta última relação: ( )Não ( ) Sim **Uso de camisinha:** ( ) Em todas ( ) Nunca ( ) As vezes

**Possui parceiro fixo?** ( ) Não ( ) Sim **Aborto**: ( ) Não ( ) Sim

**Qual método contraceptivo você utiliza**: ( )Preservativo ( )DIU ( ) Pílula ( ) Outro ( ) Não usa

**Idade do primeiro parto Possui filhos?** ( ) Não ( ) Sim **Quantos**?

Nº de partos normais:

**Higiene íntima**: **Externa** ( ) Não ( ) Sim **Interna** ( ) Não ( ) Sim, qual?

**Terapia de reposição hormonal**: ( ) Não ( ) Sim **Período da terapia**:

**Faz exame preventivo ginecológico anualmente?** ( ) Não ( ) Sim ( ) Primeira coleta

Em que ano fez o último exame preventivo (PCCU)? onde fez? Já teve câncer? ( ) Não ( ) Sim Qual/em qual parte do corpo?

Faz uso de algum medicamento, (inclusive antibiótico) nas últimas 72h? ( ) Não ( ) Sim Qual (is)?

**Toma remédio para verme**: ( ) Não ( ) Sim **quando (mês ou ano): fez segunda dose?** ( ) Não ( ) Sim

**Apresenta algum problema ginecológico?** ( )Corrimento ( )Sangramento ( )Dores ( ) Ardência ao urinar ( ) Cheiro forte ( ) Prurido/coceira ( ) outros ( ) Não apresenta

**Uso de drogas:** ( )Não ( )Sim **Qual? ultimo ano de parou: Já teve alguma DST?** ( )Não ( )Sim **Qual?**

# 1
