# Supplementary material for: High prevalence of sexual infection by human papillomavirus and Chlamydia trachomatis in sexually-active women from a large city in the Amazon region of Brazil
Source: PLoS One. 2022 Jul 18;17(7):e0270874. doi: 10.1371/journal.pone.0270874 (PMC9292084; doi:10.1371/journal.pone.0270874)
Supplement: S3 File — (DOCX) [file pone.0270874.s003.docx]

[
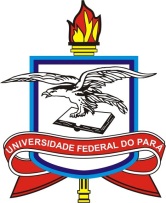
](http://www.google.com.br/url?sa=i&rct=j&q=UFPA&source=images&cd=&cad=rja&docid=jkIEGu5gAotnsM&tbnid=wjbu2gWw3AD0XM:&ved=0CAUQjRw&url=http://pt.wikipedia.org/wiki/Ficheiro:Brasao_UFPA.jpg&ei=5SUeUt3YLoGs9ASA54GIBg&bvm=bv.51156542,d.eWU&psig=AFQjCNEwacBSVUTrrAnT34jmTl_ox4_hKQ&ust=1377793843717452)

SERVIÇO PÚBLICO FEDERAL

UNIVERSIDADE FEDERAL DO PARÁ

NÚCLEO DE MEDICINA TROPICAL

PROGRAMA DE PÓS-GRADUAÇÃO EM DOENÇAS TROPICAIS

**TERMO DE CONSENTIMENTO LIVRE E ESCLARECIDO**

**Projeto: “DIVERSIDADE GENOTÍPICA DE *Chlamydia trachomatis* EM INFECÇÃO SEXUAL EM UNIVERSITÁRIAS DO ESTADO DO PARÁ*”***

**Senhora**____________________________________________________________

Solicitamos sua participação voluntariamente nesta pesquisa que tem como objetivo INVESTIGAR E PREVENIR AS DOENÇAS SEXUALMENTE TRANSMISSÍVEIS, CAUSADAS POR BACTÉRIAS, EM MULHERES DO ESTADO DO PARÁ. Caso você aceite participar, serão coletados dois espécimes cervicais (amostras do colo do útero), um para o exame de biologia molecular (verificação de moléculas) e outro para exame citológico (observação das condições das células do colo do útero). Serão tomados todos os CUIDADOS NECESSÁRIOS para que a coleta seja MENOS INCÔMODA POSSÍVEL, pois esta será realizada por profissional capacitado, que não comentará em hipótese alguma, condições da amostra bem como da quem se submeter ao exame.

Seus RESULTADOS serão tratados de forma ANÔNIMA e CONFIDENCIAL, isto é, seus dados NÃO SERÃO DIVULGADOS em qualquer fase do estudo. Quando for necessário exemplificar determinada situação, sua privacidade será assegurada uma vez que seu nome será substituído de forma aleatória. Os dados coletados serão utilizados apenas nesta pesquisa e os resultados divulgados em eventos e/ou revistas científicas.

Sua participação neste estudo consistirá em responder as perguntas a serem realizadas sob a forma de questionário. E a doação de material biológico (secreção cérvico-vaginal). NÃO HAVERÁ NENHUM CUSTO OU QUAISQUER OU PAGAMENTOS, E SERÃO AMENIZADOS AO MÁXIMO OS RISCOS, DE QUALQUER NATUREZA, RELACIONADA A SUA PARTICIPAÇÃO. O benefício direto relacionado à sua participação será a obtenção dos resultados dos exames, além do beneficiamento à comunidade científica através dos resultados da pesquisa.

FICA CLARO que a participante da pesquisa, ou o seu representante legal, pode a qualquer momento retirar seu consentimento, não tendo nenhum tipo de ônus ou pagamento, e que sua desistência em nada compromete o seu atendimento junto aos serviços oferecidos pela equipe do projeto.


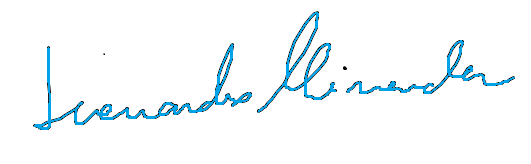
_

**Leonardo Miranda dos Santos (Pesquisador responsável)**

**Consentimento:**

**Declaro que li e compreendi as informações sobre a pesquisa, que me sinto perfeitamente esclarecido sobre o conteúdo da mesma, assim como os seus riscos e benefícios. Declaro ainda que, por minha livre vontade, aceito participar da pesquisa cooperando com a coleta de material para exame.**

**Belém, Pará, Brasil_______/________/________.**

**Assinatura da participante**

Ambulatório do Núcleo de Medicina Tropical - Av. Generalíssimo Deodoro, nº 92, Umarizal -fone: (91)3201-6812. CEP: 66055240
