# Supplementary material for: High prevalence of sexual infection by human papillomavirus and Chlamydia trachomatis in sexually-active women from a large city in the Amazon region of Brazil
Source: PLoS One. 2022 Jul 18;17(7):e0270874. doi: 10.1371/journal.pone.0270874 (PMC9292084; doi:10.1371/journal.pone.0270874)
Supplement: S2 File — (DOCX) [file pone.0270874.s002.docx]

SERVIÇO PÚBLICO FEDERAL UNIVERSIDADE FEDERAL DO PARÁ NÚCLEO DE MEDICINA TROPICAL

Belém of Pará, Brazil, 02/02/2022

Dear Dr. Leonardo Miranda dos Santos,

Our institution has always encouraged scientific publication of our teachers, however due to the economic difficulty that the country is going through, the estimated budget for this year was reduced and its transfer is coming late. Thus, unfortunately, at this moment, it is not possible for us give you the necessary support to publish your material.

We regret very much and hope your understanding.

Sincerely,

Edna Aoba Yassui Ishikawa Diretora Adjunta do NMT/UFPA

Av. Generalíssimo Deodoro, 92 – Umarizal - Cep: 66055-240- Belém-Pa. Fone: (91) 3201-0967
